# Importance of Carbohydrate Quality: What Does It Mean and How to Measure It?

**DOI:** 10.1093/jn/nxac039

**Published:** 2022-02-18

**Authors:** Vanessa Campos, Luc Tappy, Lia Bally, John L Sievenpiper, Kim-Anne Lê

**Affiliations:** Department of Nutrition Sciences, Nestlé Research, 1000 Lausanne 26, Switzerland; Department of Diabetology, Endocrinology, Nutrition & Metabolism, Inselspital, Bern, Switzerland; Department of Diabetology, Endocrinology, Nutrition & Metabolism, Inselspital, Bern, Switzerland; Departments of Nutritional Sciences and Medicine, University of Toronto, Toronto, Ontario, Canada; Division of Endocrinology & Metabolism, Department of Medicine, St. Michael's Hospital, Toronto, Ontario, Canada; Toronto 3D Knowledge Synthesis & Clinical Trials Unit, Clinical Nutrition and Risk Factor Modification Centre, St. Michael's Hospital, Toronto, Ontario, Canada; Li Ka Shing Knowledge Institute, St. Michael's Hospital, Toronto, Ontario, Canada; Department of Nutrition Sciences, Nestlé Research, 1000 Lausanne 26, Switzerland

**Keywords:** carbohydrates, nutrient profile, fibers, sugars, dietary surveys

## Abstract

Dietary carbohydrates are our main source of energy. Traditionally, they are classified based on the polymer length between simple and complex carbohydrates, which does not necessarily reflect their impact on health. Simple sugars, such as fructose, glucose, and lactose, despite having a similar energy efficiency and caloric content, have very distinct metabolic effects, leading to increased risk for various chronic diseases when consumed in excess. In addition, beyond the absolute amount of carbohydrate consumed, recent data point out that the food form or processing level can modulate both the energy efficiency and the cardiometabolic risk associated with specific carbohydrates. To account for both of these aspects—the quality of carbohydrates as well as its food form—several metrics can be proposed to help identifying carbohydrate-rich food sources and distinguish between those that would favor the development of chronic diseases and those that may contribute to prevent these. This review summarizes the findings presented during the American Society of Nutrition Satellite symposium on carbohydrate quality, in which these different aspects were presented.

## Introduction

The health effects of dietary carbohydrate have been the object of much attention. Until recently, dietary recommendations have mainly specified the proportion of desired carbohydrate in the diet and partitioned carbohydrate into complex (i.e., starch) and simple (i.e., sugars). This classification may not adequately address the health effects of sugars, however: prospective observational studies provide overwhelming evidence that sugar-containing foods such as fruit and added sugars, or some starchy foods such as whole grains, pulses, or potatoes, are associated with different effects on the risk of several noncommunicable diseases. This has led to the concept of “carbohydrate quality,” which aims to address the differing associations between carbohydrate from various food sources on health outcomes.

### Carbohydrate classification

Carbohydrates constitute one of the 3 major classes of dietary energy substrates. They are built from basic units of monosaccharide, each containing n atoms of carbons (e.g., pentoses and hexoses containing 5 and 6 carbon atoms, respectively) of which n–1 carbons carry alcohol residues and 1 carbon an aldehyde or ketone residue. Carbohydrates can further be classified according to their degree of polymerization as mono-, di-, and polysaccharides (1).

The most common monosaccharides present in the human diet are glucose and fructose contained in fruit, vegetables, honey. and natural syrups (i.e., maple syrup). The most common disaccharides include sucrose (a glucose-fructose dimer present in fruit and vegetables) and lactose (a glucose-galactose dimer present in dairy products). Sucrose can also be extracted and refined in large quantities from sugarcane or sugar beets. In addition, syrups containing glucose and fructose in various proportions can be produced industrially from starch-containing plants. The most commonly used syrup in the food supply is high-fructose corn syrup (HFCS), which is widely used, mostly in the United States (2). Mono- and disaccharides elicit a sweet taste and are commonly referred to as “sugars.” Refined sucrose, HFCS, honey, natural syrups, and fruit juice concentrates are generally added to foods during their preparation and are therefore referred to as “added” or “free” sugars, to distinguish them from sugars naturally present in fruit, vegetables, and dairy (3). Finally, polysaccharides contain 3 to several thousand monosaccharides. There is a large variety of polysaccharides in plants, but only starches, which are ramified polymers of glucose linked with α1,4 and α1,6 glycosidic bonds, are digestible by human digestive enzymes and are considered dietary carbohydrates. Nonstarch polysaccharides, containing mixtures of glucose and other monosaccharides, are not digestible by human enzymes and constitute the major source of dietary fibers. Of note, these compounds can be fermented by colonic bacteria to short-chain fatty acids and lactate, which can secondarily be absorbed in the bloodstream and metabolized by the human cells. Ingested mono- and disaccharides and starches deliver ∼4 kcal/g to humans, whereas dietary fibers are considered to deliver ∼2 kcal/g (4).

Monosaccharides, disaccharides, and starches are digested by pancreatic and intestinal enzymes in the gut and are absorbed in the bloodstream as monosaccharides (i.e., glucose, fructose, galactose). Glucose is a prime energy source for all human cells and an (almost) exclusive source of energy for the brain. However, it can be synthesized endogenously from amino acids or glycerol. Monosaccharides are also constituents of mucopolysaccharides and glycosylated proteins in human cells, but the specific monosaccharides required for these processes can be synthesized endogenously. Monosaccharides, disaccharides, and starches are therefore not considered essential nutrients, and their only physiologic function is energy provision to the cells (5). However, carbohydrates still represent a major portion of the energy present in available foods, therefore making a major contribution to energy intake when part of a varied diet (6).

### Carbohydrate quality from a physiologic perspective

The quality of a nutrient from a physiologic perspective may be defined according to the way it fulfills its physiologic role (i.e., how efficiently it transfers energy to the cells of the organism) and, according to the absence of adverse health effects, is associated with its intake. The latter is relevant given that cardiovascular and metabolic diseases contribute substantially to the global burden of disease and are primarily driven by environmental factors, including nutrition (7). Thus, adverse health effects of a specific nutrient may be unraveled by studying how its consumption influences major cardiometabolic risk markers such as body weight, glucose homeostasis, insulin sensitivity, or blood lipid profiles.

#### Energy efficiency

Energy efficiency of carbohydrate can be defined as the ratio of the energy used by cells (as chemical or mechanical work) to the energy content of the initial food (predigestion) (8). The main factors responsible for lowering this ratio, and hence lowering the energy efficiency of a given carbohydrate, include incomplete digestion or absorption and loss of energy to heat during the process of thermogenesis (9). The monosaccharide glucose does not require digestion and is entirely absorbed from the gut by an energy-requiring sodium-glucose cotransporter. Furthermore, blood glucose is a prime energy substrate readily used by all cells of the organism with little energy loss (needed to initially activate glucose to fructose 1,6 diphosphate prior to further degradation to CO_2_ and H_2_O) and will be taken as a reference here. The disaccharides sucrose and lactose are normally completely digested by gut disaccharidases and absorbed into the bloodstream as glucose, galactose, and fructose. In adults, however, lactase deficiency is present in a substantial portion of the population, resulting in incomplete digestion of lactose (10). Galactose is completely absorbed by the same sodium-glucose cotransporter as glucose. It is, however, converted to glucose-1-phosphate and glycogen in the liver before being released into the circulation as glucose, leading to an energy loss of ∼2% (11). In contrast, fructose is absorbed from the gut through simple, facilitated diffusion and is therefore incomplete, particularly when ingested in large amounts (12). It is initially converted into triose phosphates in the enterocytes and hepatocytes prior to being released into the bloodstream as lactate, glucose, or triglyceride, and this splanchnic metabolism accounts for the variable losses of energy (ranging from 5% when released as glucose to 25–30% when released as triglycerides) (13).

Although it is expected that the energy efficiency of starch would be similar to that of glucose, being composed of glucose monomers, several factors, including variation in starch structure according to its plant origin and food processing, result in differences in its energy availability (14). Furthermore, the digestion of starch is facilitated by swelling of starch granules produced during cooking, which slows digestion and absorption, but this phenomenon varies among various starchy foods, with lesser swelling and slower digestion and absorption of starch from unprocessed cereals than from refined cereal products (15).

#### Effects on cardiometabolic risk factors

A high dietary intake of carbohydrates is associated with an increase in total and VLDL triglycerides and a decrease in HDL cholesterol concentrations (16), which are recognized risk factors for the development of cardiovascular diseases. The adverse effects of a high carbohydrate intake have been attributed to glucose-induced hyperinsulinemia and stimulation of de novo lipogenesis and of VLDL-triglyceride secretion from the liver (16, 17). Compared with dietary starch or glucose, which are only partially metabolized in the liver, fructose is efficiently taken up by hepatocytes, where it stimulates gluconeogenesis and lipogenesis. This is associated with increased intrahepatic fat concentrations, enhanced hepatic VLDL-triglyceride secretion, and impaired suppression of hepatic glucose production. These effects may hypothetically proceed toward the development of nonalcoholic fatty liver disease, insulin resistance and diabetes mellitus, and atherosclerosis (18). Of interest, these deleterious effects of fructose can be blunted when physical activity is performed right after fructose ingestion, by immediately increasing fructose oxidation and decreasing fructose storage (19). This suggests that deleterious effects of fructose and fructose-containing sugars can be compensated by a high whole-body energy output associated with high physical activity level (19). Although the effects of galactose and lactose on these parameters have not been comprehensively studied, a recent study reports that dietary galactose may increase blood triglyceride concentration to the same extent as fructose (20). Finally, some alternative sugars, although sometimes classified as “added sugars,” may have distinct physiologic effects. Isomaltulose, an isomer of sucrose with glucose and fructose linked through an α-1,6 linkage instead of an α-1,2 linkage in sucrose, is digested at a slower rate than sucrose due to lower intestinal enzyme affinity, thus lowering postprandial glucose response and related inflammatory markers in patients with type 2 diabetes (21). Among other rare sugars, allulose is a C3 epimer of D-fructose and tagatose a stereoisomer of D-fructose (22). Due to their different conformations, they are either not metabolized (in the case of allulose) or not absorbed (in the case of tagatose), which results in a lower caloric content for both rare sugars. Beyond their lower caloric content, some studies have suggested they may have additional benefits by decreasing glucose-induced postprandial glycemia. The postulated mechanism may involve inhibition of α-glucosidase (22). The associations between total dietary carbohydrate intake and health-related outcomes have been intensively studied but remain debated. Recent evidence from the PURE (Prospective Urban Rural Epidemiology) study, which assessed the relation between self-reported carbohydrate, fat, and protein intake from >130,000 participants from 18 countries, concluded that high carbohydrate intake was associated with higher risk of total and cardiovascular mortality, whereas total fat and individual types of fat were related to lower risks (23). These conclusions of a negative effect of a diet high in carbohydrates are, however, not universally applicable. Of note, there is substantial evidence that consumption of a hypocaloric diet results in weight loss proportionate to energy deficit irrespective of the total dietary carbohydrate and fat content (24), suggesting that a high-carbohydrate diet may not be a major factor promoting weight gain and associated cardiovascular comorbidity.

### Role of processing and food forms

Beyond the absolute amount of carbohydrates consumed, there is now compelling evidence that the associations between carbohydrate intake and health-related outcomes vary according to dietary carbohydrate sources. Systematic reviews and meta-analyses of prospective cohort studies clearly indicate that carbohydrates from fruit, pulses, or whole-grain products are associated with lower mortality and risk of cardiometabolic diseases, whereas refined carbohydrates and added sugars (more specifically from sugar-sweetened beverages) are associated with higher risks (25). These observations are further corroborated by meta-analyses of intervention studies showing decreased risk of cardiometabolic diseases when dietary intake of whole grain (26, 27), pulses (28–31), or fruit (32, 33) is increased.

The underlying mechanisms responsible for differences in health effects across carbohydrate-containing foods remain incompletely understood. One possible explanation may be the differences in postprandial blood glucose excursions and insulin responses elicited by different types of carbohydrate. According to this hypothesis, carbohydrates that cause a rapid rise in blood glucose (high glycemic index) may be associated with early postprandial hyperinsulinemia favoring energy storage and late postprandial hypoinsulinemia triggering food intake, whereas these effects would not be observed with low glycemic index carbohydrate. This hypothesis is supported by prospective cohort studies showing that consumption of a high glycemic index diet is associated with increased incidence of diabetes and cardiovascular diseases (34–36). The lower glycemic impact of carbohydrates may be related to a slower rate of intestinal glucose absorption or hepatic first-pass metabolism (i.e., fructose or galactose) and often corresponds to foods high in dietary fiber content. Dietary fibers slow down gastrointestinal transit time and may therefore delay dietary carbohydrate absorption. In addition, they exert beneficial effects on gut microbiota and are fermented by gut bacteria, thus producing short-chain fatty acids. These microbiota-generated metabolites promote beneficial effects on colonocyte health as well as metabolic health and energy homeostasis (e.g., via satiety signals in the brain) of the host. Systematic reviews of prospective cohort studies indeed indicate that total dietary fiber intake is associated with a lower risk of diabetes (37) or cardiovascular diseases (38). Intervention studies also indicate that supplementation with viscous fibers such as β-glucan, psyllium, or guar gum improve glucose control in patients with diabetes (39) and lower blood cholesterol (40) and systolic blood pressure (41) in the general population. Finally, the glycemic impact of carbohydrates is further influenced by their interactions with other macronutrients present in food (e.g., the matrix effect). For example, glucose combined with protein or fat provides differing impacts on glucose and insulin excursions compared with those exhibited when consumed in isolation (42).

Some studies have indicated that the source of dietary fibers may play a role in the prevention of chronic diseases, with cereal fibers showing the strongest association with risk reduction of type 2 diabetes and cardiovascular diseases (43, 44). Beyond the food sources, and similarly to starch, there is also evidence that the degree of processing can alter the fiber's beneficial effects on health (45). Harsh mechanical and/or thermal treatments used in some food processing can disrupt the fiber network and physicochemical structures, thus modifying the way they interact with digestible carbohydrates present in whole foods (46).

### Pragmatic metrics to help define carbohydrate quality

Based on physiologic considerations and on human prospective and intervention studies, there is clear evidence that carbohydrate quality differs across various foods and has highly relevant importance for human health. It can be readily communicated for traditional, unprocessed, or minimally processed foods such as whole grains, fresh fruit, pulses, and sugars (39). When it comes to packaged or ready-to-eat foods, however, a clear, multidimensional definition of carbohydrate quality is required.

A recent article, reporting on several meta-analyses from prospective cohort studies and intervention studies, identified whole grains and dietary fiber as major food components related to beneficial health outcomes (25). Whole grain and products made from it are defined by the Oldways Whole Grain Council as products containing “all the essential parts and naturally-occurring nutrients of the entire grain seed in their original proportions” (47). There are, however, various alternative definitions of whole grain that may lead to some degree of confusion (48). In addition, consumption of added or free sugars is increasingly recognized as being associated with adverse health effects (49). It appears, therefore, that a high dietary fiber content together with a low added sugar content would be key descriptors of carbohydrate quality. The WHO dietary guidelines indeed recommend daily consumption of >25 g dietary fiber and that free sugars represent <10% of total energy (49). These recommendations are only met by a minority of the Australian population (50–52). In the US population, according to NHANES 2015–2016, carbohydrates account for ∼52% of total daily energy intake. Of these, only 9% are from high-quality carbohydrates such as whole grain, fruit, and pulses, while the vast majority was from refined grain, starchy vegetables, added sugars, and sweet beverages (53).

From a practical point of view, one major challenge imposed on nutritionists is how to communicate to the general population the best and simplest way of identifying high-quality carbohydrates. This is particularly relevant for industrial products, the composition of which is not immediately apparent to the consumer. Based on the associations observed between added sugar and dietary fiber intakes and health outcomes, several empirical indexes reflecting carbohydrate quality of carbohydrate-rich, packaged food products have been proposed. The simplest one is a total carbohydrate to dietary fiber ratio of <10:1 to target fiber-rich carbohydrate products. Modified forms of this index, including a total carbohydrate (g) to dietary fiber (g) ratio of <10:1, together with a total carbohydrate (g) to added sugar of <10:1 or 10:2, have therefore been developed (**Table 1**) (54).

**TABLE 1:** Carbohydrate quality ratios

Ratio	Definition
10:1 carb:fiber ratio	Original validated ratio defined as ≥ 1 g of fiber per 10 g of carbohydrate
10:1:1 carb:fiber:added sugars ratio	Ratio defined as ≥ 1 g of fiber and < 1 g of added sugars per 10 g of carbohydrate
10:1:2 carb:fiber:added sugars ratio	Ratio defined as ≥ 1 g of fiber and < 2 g of added sugars per 10 g of carbohydrate
10:1|1:2 carb:fiber and fiber:added sugars ratio	Ratio defined as ≥ 1 g of fiber per 10 g of carbohydrate and < 2 g of added sugars per 1 g of fiber

Table adapted from (54).

A recent study calculated the composition of carbohydrates containing products meeting the 10:1 carbohydrate/fiber ratio consumed by urban residents of São Paulo, Brazil. These products contained less added sugar and saturated fat and more dietary fiber and protein per serving than products with a carbohydrate/fiber ratio >10:1. In addition, they also contained more micronutrients (potassium, magnesium, selenium, zinc) per serving. Furthermore, in this population, consumption of food products meeting the 10:1 carbohydrate/fiber ratio was associated with decreased blood triglyceride concentration, lower fasting insulin concentration, and better indexes of insulin sensitivity. Application of the ≤10:1 carbohydrate to fiber ratio was shown to identify healthy grain foods and their association with cardiometabolic risk factors (55). In Australia, according to the 2013–2016 nutrition survey, the 10:1 ratio was attained by 50% of products consumed by adults and by 29% of those consumed by children, whereas the modified 10:1:2 ratio was attained in only 33% and 19% and the dual ratio in 41% and 22%, respectively (56). Products meeting any of these indexes of quality contained on average more energy, protein, unsaturated fat, B-vitamins (with the exception of vitamin B-12), iron, magnesium, and zinc and less total carbohydrate, added sugars, and sodium than products that did not meet any index of quality, indicating a globally higher nutritional value (51). Interestingly, a recent study showed that the 10:1 ratio proved to be applicable to beverages, showing that increasing the fiber content allows differentiation of the more nutritious products with a higher content of proteins and dietary fibers, as well as lower caloric content, added sugars, cholesterol, and sodium (57). These results demonstrated that this metric serves as an effective method to identify foods with a better nutritional quality. This is further supported by a recent study that confirmed that these metrics are associated with improved diet quality (58). Diets meeting the original validated 10:1 carbohydrate/fiber ratio, the modified 10:1:2 carbohydrate/fiber/added sugars ratio, and the dual 10:1 and 1:2 carbohydrate/fiber and fiber/added sugars ratio 10:1 and 1:2 had lower energy, total sugar, and saturated fat intakes but higher protein, monounsaturated and polyunsaturated fatty acids, and dietary fiber compared with those that did not meet the ratios. Furthermore, diets meeting the target ratios were characterized by higher daily intakes of several micronutrients (B-vitamins, vitamin E, folate, iron, magnesium, zinc) and higher Healthy Eating Index for Australian Adults (2013) with lower intakes of discretionary foods/beverages (high in saturated fat and/or added sugars, added salt, or alcohol) and higher intakes of vegetables, fruit, grains, meat, water, fat, sodium, and added sugars. The same report also describes a novel dietary modeling analysis examining the impact of consuming foods that satisfy the carbohydrate ratios on estimated nutrient intakes. The authors replaced in the model each carbohydrate-based food that did not satisfy a ratio with the closest foods that met the carbohydrate ratio. This substitution analysis resulted in an increased energy, protein, total fat, mono- and polyunsaturated fatty acid, and dietary fiber intake but less total carbohydrate, added sugar, and free sugar. Intake of micronutrients increased with diet modeling analysis but interestingly did not improve the intakes of folate and vitamin B-12. A possible explanation provided by the authors may be local fortification rules.

**FIGURE 1:**
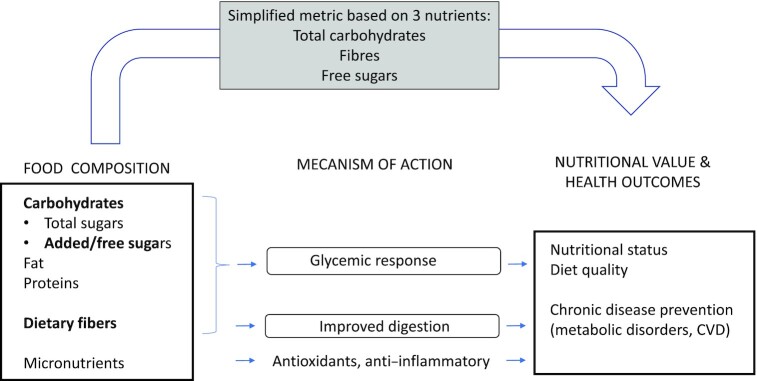
Proposed new carbohydrates metrics to reflect food composition and its impact on health outcomes: The nutritional quality of a product is reflected by the sum and interaction of its individual nutrients, including macro- and micronutrients. A simplified extract of this full nutritional composition can be represented by a metric taking into account only three nutrients: total carbohydrates, fibres and added sugars. Products compliant with such metric were showed have higher nutritional quality, which may positively impact diet quality and health outcomes. CVD, cardiovascular disease.

Existing markers of carbohydrate quality in a product include the glycemic index, whole-grain claims, and avoidance of added sugar. Although the glycemic index is well recognized in a limited number of countries (e.g., Nordic countries and Italy), its use beyond these countries is presently limited and requires clinical tests to determine its value (59). Presence of a whole-grain claim in a food ensures that a minimum requirement for whole-grain content is met but, as mentioned above, does not guarantee that the product meets other basics nutrition requirements, such as an upper limit for added sugars (48). The ratio mentioned in the present article can to some extent overcome these issues and provide a complementary approach, as it can be easily assessed from nutritional facts and ensures in most cases that the food products have superior nutritional quality (**Figure 1**). With respect to added sugar, there are currently limitations of developing food databases with information on added sugar and front-of-package labels indicating added sugar content remain scarce (60). The ratio has been primarily developed to help discriminate the nutritional quality of cereal-based products (54, 61). This was initially thought to provide some guardrail for the application of the ratio to pure sugar-based confectionary products, for example, where the addition of fibers may not be enough to qualify such products as “healthy,” or products primarily high in proteins or fat, for which such a ratio may not be relevant. However, it may be considered to extend the use of such a ratio beyond cereals, to legume-, nut-, and seed-based products, as these foods are of high nutritional value and can positively contribute to reaching the ratio by providing a good fiber source.

Despite the fact that this approach, based on total carbohydrate to dietary fiber and added sugars ratios, is empirical and based on only 3 nutrients, it allows discriminating food products of higher nutritional value. The beneficial effects of foods may be linked not only to their fiber and/or total sugar content but also to the presence of other compounds present in whole grains, pulses, and fruit, such as vitamins, micronutrients, or polyphenols. As such, these different ratios based on total carbohydrates, added sugars, and dietary fibers may indirectly reflect foods’ global nutrients content and hence nutritional value.

In conclusion, there is increasing evidence that the effects of carbohydrate-containing foods on health-related outcomes vary widely according to the food sources, a phenomenon referred to as “carbohydrate quality.” There is strong evidence that consumption of whole-grain products and a high dietary fiber intake are associated with benefits, whereas consumption of added sugars and refined carbohydrates is linked with adverse health outcomes. Based on these observations, various empirical indexes based on the ratio of total carbohydrate to dietary fiber and added sugar contents reflecting carbohydrate quality have been proposed. Among these, a simple ratio of total carbohydrate/dietary fiber <10:1 appears to be the best suited in promoting the choice of healthier carbohydrate sources. All these indexes identified products with higher protein, vitamins, and micronutrient contents and hence with higher overall nutritional qualities. Future studies are still needed to test the use of these ratios for consumer messaging or policy actions (e.g., as part of front-of-package labeling systems, nutrition facts, and/or nutrition targets for industry).

## Notes

This review summarizes the content of a symposium held during the Annual American Nutrition Society conference, sponsored by Nestlé Research. The funder had no influence on the symposium's content. JLS was funded by a Diabetes Canada Clinician Scientist Award.

Author disclosures: VC and K-AL are employed by Nestlé Research, Switzerland. LT has received speaker fees from Soremartec Italy srl and consulting fees from Nestlé SA Switzerland. JLS has received research support from the Canadian Foundation for Innovation, Ontario Research Fund, Province of Ontario Ministry of Research and Innovation and Science, Canadian Institutes of Health Research, Diabetes Canada, PSI Foundation, Banting and Best Diabetes Centre, American Society for Nutrition (ASN), INC International Nut and Dried Fruit Council Foundation, National Dried Fruit Trade Association, National Honey Board (the USDA honey “Checkoff” program), International Life Sciences Institute (ILSI), Pulse Canada, Quaker Oats Center of Excellence, United Soybean Board (the USDA soy “Checkoff” program), Tate and Lyle Nutritional Research Fund at the University of Toronto, Glycemic Control and Cardiovascular Disease in Type 2 Diabetes Fund at the University of Toronto (a fund established by the Alberta Pulse Growers), and Nutrition Trialists Fund at the University of Toronto (a fund established by an inaugural donation from the Calorie Control Council). He has received in-kind food donations to support a randomized controlled trial from the Almond Board of California, California Walnut Commission, Peanut Institute, Barilla, Unilever/Upfield, Unico/Primo, Loblaw Companies, Quaker, Kellogg Canada, WhiteWave Foods/Danone, and Nutrartis. He has received travel support, speaker fees, and/or honoraria from Diabetes Canada, Dairy Farmers of Canada, FoodMinds LLC, International Sweeteners Association, Nestlé, Pulse Canada, Canadian Society for Endocrinology and Metabolism, GI Foundation, Abbott, General Mills, Biofortis, ASN, Northern Ontario School of Medicine, INC Nutrition Research & Education Foundation, European Food Safety Authority, Comité Européen des Fabricants de Sucre, Nutrition Communications, International Food Information Council, Calorie Control Council, and Physicians Committee for Responsible Medicine. He has or has had ad hoc consulting arrangements with Perkins Coie LLP, Tate & Lyle, Wirtschaftliche Vereinigung Zucker e.V., Danone, and Inquis Clinical Research. He is a member of the European Fruit Juice Association Scientific Expert Panel and former member of the Soy Nutrition Institute Scientific Advisory Committee. He is on the Clinical Practice Guidelines Expert Committees of Diabetes Canada, European Association for the Study of Diabetes (EASD), Canadian Cardiovascular Society, and Obesity Canada/Canadian Association of Bariatric Physicians and Surgeons. He serves or has served as an unpaid scientific advisor for the Food, Nutrition, and Safety Program and the Technical Committee on Carbohydrates of ILSI North America. He is a member of the International Carbohydrate Quality Consortium, executive board member of the Diabetes and Nutrition Study Group of the EASD, and director of the Toronto 3D Knowledge Synthesis and Clinical Trials foundation. His wife is an employee of AB InBev. LB declares no conflict of interest related to this work.

## References

[1] Cummings JH , Roberfroid MB, Andersson H, Barth C, Ferro-Luzzi A, Ghoos Y, Gibney M, Hermansen K, James WP, Korver O et al. A new look at dietary carbohydrate: chemistry, physiology and health. Paris Carbohydrate Group. Eur J Clin Nutr. 1997;51(7):417–23. 9234022 10.1038/sj.ejcn.1600427

[2] Hanover LM , White JS. Manufacturing, composition, and applications of fructose. Am J Clin Nutr. 1993;58(5):724S–32S. 8213603 10.1093/ajcn/58.5.724S

[3] Marinho AR , Severo M, Correia D, Lobato L, Vilela S, Oliveira A, Ramos E, Torres D, Lopes C. Total, added and free sugar intakes, dietary sources and determinants of consumption in Portugal: the National Food, Nutrition and Physical Activity Survey (IAN-AF 2015–2016). Public Health Nutr. 2020;23(5):869–81. 31486357 10.1017/S1368980019002519 PMC10200657

[4] Institute of Medicine . Dietary reference intakes for energy, carbohydrate, fiber, fat, fatty acids, cholesterol, protein, and amino acids. Washington (DC): The National Academies Press; 2005.

[5] Harper A . Defining the essentiality of nutrients. In: Shils M, Olson J, Shihe M, AC Reditors. Modern nutrition in health and disease. Boston (MA): William & Wilkins; 1999. p. 3–10.

[6] Jéquier E . Carbohydrates as a source of energy. Am J Clin Nutr. 1994;59(3):682S–5S. 8116550 10.1093/ajcn/59.3.682S

[7] GBD 2019 Risk Factors Collaborators . Global burden of 87 risk factors in 204 countries and territories, 1990–2019: a systematic analysis for the Global Burden of Disease Study 2019. Lancet. 2020;396(10258):1223–49. 33069327 10.1016/S0140-6736(20)30752-2 PMC7566194

[8] Hall KD , Heymsfield SB, Kemnitz JW, Klein S, Schoeller DA, Speakman JR. Energy balance and its components: implications for body weight regulation. Am J Clin Nutr. 2012;95(4):989–94. 22434603 10.3945/ajcn.112.036350 PMC3302369

[9] Acheson K , Jéquier E, Burger A, Danforth E Jr. Thyroid hormones and thermogenesis: the metabolic cost of food and exercise. Metabolism. 1984;33(3):262–5. 6694567 10.1016/0026-0495(84)90048-9

[10] Montalto M , Curigliano V, Santoro L, Vastola M, Cammarota G, Manna R, Gasbarrini A, Gasbarrini G. Management and treatment of lactose malabsorption. World J Gastroenterol. 2006;12(2):187–91. 16482616 10.3748/wjg.v12.i2.187 PMC4066025

[11] Hui YS , Bin LLW, Foong-Fong MC. Metabolism of simple sugars. In: Goran M, Tappy L, Lê K-Aeditors. Dietary sugars and health. Boca Raton (FL): CRC Press; 2015. p. 157–68.

[12] Ferraris RP , Choe JY, Patel CR. Intestinal absorption of fructose. Annu Rev Nutr. 2018;38(1):41–67. 29751733 10.1146/annurev-nutr-082117-051707 PMC6457363

[13] Tappy L , Egli L, Lecoultre V, Schneider P. Effects of fructose-containing caloric sweeteners on resting energy expenditure and energy efficiency: a review of human trials. Nutr Metab. 2013;10:54. 10.1186/1743-7075-10-54 PMC3751443 23941499

[14] Englyst KN , Liu S, Englyst HN. Nutritional characterization and measurement of dietary carbohydrates. Eur J Clin Nutr. 2007;61(Suppl 1):S19–39. 17992185 10.1038/sj.ejcn.1602937

[15] Englyst HN , Kingman SM, Hudson GJ, Cummings JH. Measurement of resistant starch in vitro and in vivo. Br J Nutr. 1996;75(5):749–55. 8695601 10.1079/bjn19960178

[16] Parks EJ , Hellerstein MK. Carbohydrate-induced hypertriacylglycerolemia: historical perspective and review of biological mechanisms. Am J Clin Nutr. 2000;71(2):412–33. 10648253 10.1093/ajcn/71.2.412

[17] Ludwig DS , Hu FB, Tappy L, Brand-Miller J. Dietary carbohydrates: role of quality and quantity in chronic disease. BMJ. 2018;361:k2340. 29898880 10.1136/bmj.k2340 PMC5996878

[18] Tappy L , Lê KA. Health effects of fructose and fructose-containing caloric sweeteners: where do we stand 10 years after the initial whistle blowings?. Curr Diab Rep. 2015;15(8):54. 26104800 10.1007/s11892-015-0627-0 PMC4477723

[19] Egli L , Lecoultre V, Theytaz F, Campos V, Hodson L, Schneiter P, Mittendorfer B, Patterson BW, Fielding BA, Gerber PA et al. Exercise prevents fructose-induced hypertriglyceridemia in healthy young subjects. Diabetes. 2013;62(7):2259–65. 23674606 10.2337/db12-1651 PMC3712038

[20] Watkins J , Simpson A, Betts JA, Thompson D, Holliday A, Deighton K, Gonzalez JT. Galactose ingested with a high-fat beverage increases postprandial lipemia compared with glucose but not fructose ingestion in healthy men. J Nutr. 2020;150(7):1765–72. 32297937 10.1093/jn/nxaa105 PMC7330468

[21] Campbell MD , Walker M, Trenell MI, Stevenson EJ, Turner D, Bracken RM, Shaw JA, West DJ. A low-glycemic index meal and bedtime snack prevents postprandial hyperglycemia and associated rises in inflammatory markers, providing protection from early but not late nocturnal hypoglycemia following evening exercise in type 1 diabetes. Diabetes Care. 2014;37(7):1845–53. 24784832 10.2337/dc14-0186

[22] Smith A , Avery A, Ford R, Yang Q, Goux A, Mukherjee I, Neville DCA, Jethwa PH. Rare sugars: metabolic impacts and mechanisms of action—a scoping review. Br J Nutr. 2021;1–77. 10.1017/S0007114521003524 PMC9343225 34505561

[23] Dehghan M , Mente A, Zhang X, Swaminathan S, Li W, Mohan V, Iqbal R, Kumar R, Wentzel-Viljoen E, Rosengren A et al. Associations of fats and carbohydrate intake with cardiovascular disease and mortality in 18 countries from five continents (PURE): a prospective cohort study. Lancet. 2017;390(10107):2050–62. 28864332 10.1016/S0140-6736(17)32252-3

[24] Ge L , Sadeghirad B, Ball GDC, da Costa BR, Hitchcock CL, Svendrovski A, Kiflen R, Quadri K, Kwon HY, Karamouzian M et al. Comparison of dietary macronutrient patterns of 14 popular named dietary programmes for weight and cardiovascular risk factor reduction in adults: systematic review and network meta-analysis of randomised trials. BMJ. 2020;370:m3095. 32238384 10.1136/bmj.m696 PMC7190064

[25] Reynolds A , Mann J, Cummings J, Winter N, Mete E, Te Morenga L. Carbohydrate quality and human health: a series of systematic reviews and meta-analyses. Lancet. 2019;393(10170):434–45. 30638909 10.1016/S0140-6736(18)31809-9

[26] Wang W , Li J, Chen X, Yu M, Pan Q, Guo L. Whole grain food diet slightly reduces cardiovascular risks in obese/overweight adults: a systematic review and meta-analysis. BMC Cardiovasc Disord. 2020;20(1):82. 32070285 10.1186/s12872-020-01337-z PMC7027052

[27] Reynolds AN , Akerman AP, Mann J. Dietary fibre and whole grains in diabetes management: systematic review and meta-analyses. PLoS Med. 2020;17(3):e1003053. 32142510 10.1371/journal.pmed.1003053 PMC7059907

[28] Kim SJ , de Souza RJ, Choo VL, Ha V, Cozma AI, Chiavaroli L, Mirrahimi A, Blanco Mejia S, Di Buono M, Bernstein AM et al. Effects of dietary pulse consumption on body weight: a systematic review and meta-analysis of randomized controlled trials. Am J Clin Nutr. 2016;103(5):1213–23. 27030531 10.3945/ajcn.115.124677

[29] Ha V , Sievenpiper JL, de Souza RJ, Jayalath VH, Mirrahimi A, Agarwal A, Chiavaroli L, Mejia SB, Sacks FM, Di Buono M et al. Effect of dietary pulse intake on established therapeutic lipid targets for cardiovascular risk reduction: a systematic review and meta-analysis of randomized controlled trials. Can Med Assoc J. 2014;186(8):E252–62. 24710915 10.1503/cmaj.131727 PMC4016088

[30] Jayalath VH , de Souza RJ, Sievenpiper JL, Ha V, Chiavaroli L, Mirrahimi A, Di Buono M, Bernstein AM, Leiter LA, Kris-Etherton PM et al. Effect of dietary pulses on blood pressure: a systematic review and meta-analysis of controlled feeding trials. Am J Hypertens. 2014;27(1):56–64. 24014659 10.1093/ajh/hpt155 PMC5391775

[31] Viguiliouk E , Glenn AJ, Nishi SK, Chiavaroli L, Seider M, Khan T, Bonaccio M, Iacoviello L, Mejia SB, Jenkins DJA et al. Associations between dietary pulses alone or with other legumes and cardiometabolic disease outcomes: an umbrella review and updated systematic review and meta-analysis of prospective cohort studies. Adv Nutr. 2019;10(Suppl 4):S308–19. 31728500 10.1093/advances/nmz113 PMC6855952

[32] Huang H , Chen G, Liao D, Zhu Y, Xue X. Effects of berries consumption on cardiovascular risk factors: a meta-analysis with trial sequential analysis of randomized controlled trials. Sci Rep. 2016;6(1):23625. 27006201 10.1038/srep23625 PMC4804301

[33] Choo VL , Viguiliouk E, Blanco Mejia S, Cozma AI, Khan TA, Ha V, Wolever TMS, Leiter LA, Vuksan V, Kendall CWC et al. Food sources of fructose-containing sugars and glycaemic control: Systematic review and meta-analysis of controlled intervention studies. BMJ. 2018;363:k4644. 30463844 10.1136/bmj.k4644 PMC6247175

[34] Livesey G , Taylor R, Livesey HF, Buyken AE, Jenkins DJA, Augustin LSA, Sievenpiper JL, Barclay AW, Liu S, Wolever TMS et al. Dietary glycemic index and load and the risk of type 2 diabetes: a systematic review and updated meta-analyses of prospective cohort studies. Nutrients. 2019;11(6):1280. 10.3390/nu11061280 PMC6627334 31195724

[35] Livesey G , Taylor R, Livesey HF, Buyken AE, Jenkins DJA, Augustin LSA, Sievenpiper JL, Barclay AW, Liu S, Wolever TMS et al. Dietary glycemic index and load and the risk of type 2 diabetes: assessment of causal relations. Nutrients. 2019;11(6):1436. 10.3390/nu11061436 PMC6628270 31242690

[36] Livesey G , Livesey H. Coronary heart disease and dietary carbohydrate, glycemic index, and glycemic load: dose-response meta-analyses of prospective cohort studies. Mayo Clin Proc Innov Qual Outcomes. 2019;3:52–69. 30899909 10.1016/j.mayocpiqo.2018.12.007 PMC6410335

[37] InterAct C . Dietary fibre and incidence of type 2 diabetes in eight European countries: the EPIC-InterAct study and a meta-analysis of prospective studies. Diabetologia. 2015;58:1394–408. 26021487 10.1007/s00125-015-3585-9 PMC4472947

[38] Threapleton DE , Greenwood DC, Evans CE, Cleghorn CL, Nykjaer C, Woodhead C, Cade JE, Gale CP, Burley VJ. Dietary fibre intake and risk of cardiovascular disease: systematic review and meta-analysis. BMJ. 2013;347:f6879. 24355537 10.1136/bmj.f6879 PMC3898422

[39] Jovanovski E , Khayyat R, Zurbau A, Komishon A, Mazhar N, Sievenpiper JL, Blanco Mejia S, Ho HVT, Li D, Jenkins AL et al. Should viscous fiber supplements be considered in diabetes control? Results from a systematic review and meta-analysis of randomized controlled trials. Diabetes Care. 2019;42(5):755–66. 30617143 10.2337/dc18-1126

[40] Brown L , Rosner B, Willett WW, Sacks FM. Cholesterol-lowering effects of dietary fiber: a meta-analysis. Am J Clin Nutr. 1999;69(1):30–42. 9925120 10.1093/ajcn/69.1.30

[41] Khan K , Jovanovski E, Ho HVT, Marques ACR, Zurbau A, Mejia SB, Sievenpiper JL, Vuksan V. The effect of viscous soluble fiber on blood pressure: a systematic review and meta-analysis of randomized controlled trials. Nutr Metab Cardiovasc Dis. 2018;28(1):3–13. 29153856 10.1016/j.numecd.2017.09.007

[42] Frid AH , Nilsson M, Holst JJ, Björck IM. Effect of whey on blood glucose and insulin responses to composite breakfast and lunch meals in type 2 diabetic subjects. Am J Clin Nutr. 2005;82(1):69–75. 16002802 10.1093/ajcn.82.1.69

[43] AlEssa HB , Bhupathiraju SN, Malik VS, Wedick NM, Campos H, Rosner B, Willett WC, Hu FB. Carbohydrate quality and quantity and risk of type 2 diabetes in US women. Am J Clin Nutr. 2015;102(6):1543–53. 26537938 10.3945/ajcn.115.116558 PMC4658465

[44] AlEssa HB , Cohen R, Malik VS, Adebamowo SN, Rimm EB, Manson JE, Willett WC, Hu FB. Carbohydrate quality and quantity and risk of coronary heart disease among US women and men. Am J Clin Nutr. 2018;107(2):257–67. 29529162 10.1093/ajcn/nqx060 PMC6454480

[45] Augustin LSA , Aas AM, Astrup A, Atkinson FS, Baer-Sinnott S, Barclay AW, Brand-Miller JC, Brighenti F, Bullo M, Buyken AE et al. Dietary fibre consensus from the International Carbohydrate Quality Consortium (ICQC). Nutrients. 2020;12(9):2553. 10.3390/nu12092553 PMC7551906 32846882

[46] Henrion M , Francey C, Lê KA, Lamothe L. Cereal B-glucans: the impact of processing and how it affects physiological responses. Nutrients. 2019;11(8):1729. 10.3390/nu11081729 PMC6722849 31357461

[47] Oldways Whole Grains Council . Definition of a whole grain [Internet]. 2021; [cited 2021 Dec 19]. Available from:https://wholegrainscouncilorg/definition-whole-grain.

[48] Mozaffarian RS , Lee RM, Kennedy MA, Ludwig DS, Mozaffarian D, Gortmaker SL. Identifying whole grain foods: a comparison of different approaches for selecting more healthful whole grain products. Public Health Nutr. 2013;16(12):2255–64. 23286205 10.1017/S1368980012005447 PMC4486284

[49] WHO . Guideline: sugars intake for adults and children. Geneva (Switzerland): WHO; 2015. 25905159

[50] Fayet-Moore F , Cassettari T, Tuck K, McConnell A, Petocz P. Dietary fibre intake in Australia. Paper II: comparative examination of food sources of fibre among high and low fibre consumers. Nutrients. 2018;10(9):1223. 10.3390/nu10091223 PMC6163727 30181455

[51] Fayet-Moore F , Cassettari T, Tuck K, McConnell A, Petocz P. Dietary fibre intake in Australia. Paper I: associations with demographic, socio-economic, and anthropometric factors. Nutrients. 2018;10(5):599. 10.3390/nu10050599 PMC5986479 29751656

[52] Gupta A , Smithers LG, Braunack-Mayer A, Harford J. How much free sugar do Australians consume? Findings from a national survey. Aust N Z J Public Health. 2018;42(6):533–40. 30296823 10.1111/1753-6405.12836

[53] Shan Z , Rehm CD, Rogers G, Ruan M, Wang DD, Hu FB, Mozaffarian D, Zhang FF, Bhupathiraju SN. Trends in dietary carbohydrate, protein, and fat intake and diet quality among US adults, 1999–2016. JAMA. 2019;322(12):1178–87. 31550032 10.1001/jama.2019.13771 PMC6763999

[54] Liu J , Rehm CD, Shi P, McKeown NM, Mozaffarian D, Micha R. A comparison of different practical indices for assessing carbohydrate quality among carbohydrate-rich processed products in the US. PLoS One. 2020;15(5):e0231572. 32437371 10.1371/journal.pone.0231572 PMC7241725

[55] Fontanelli MM , Micha R, Sales CH, Liu J, Mozaffarian D, Fisberg RM. Application of the ≤10:1 carbohydrate to fiber ratio to identify healthy grain foods and its association with cardiometabolic risk factors. Eur J Nutr. 2020;59(7):3269–79. 31865421 10.1007/s00394-019-02165-4

[56] Blumfield M , McConnell A, Cassettari T, Petocz P, Warner M, Campos V, Le KA, Minehira K, Marshall S, Fayet-Moore F. Balanced carbohydrate ratios are associated with improved diet quality in Australia: a nationally representative cross-sectional study. PLoS One. 2021;16(7):e0253582. 34242252 10.1371/journal.pone.0253582 PMC8270120

[57] Tan D , Olden AN, Orengo A, Francey C, Campos VC, Fayet-Moore F, Kim JE, Le KA. An assessment of three carbohydrate metrics of nutritional quality for packaged foods and beverages in Australia and Southeast Asia. Nutrients. 2020;12(9):2771. 10.3390/nu12092771 PMC7551443 32932799

[58] Blumfield M MA , Cassettari T, Warner M, Campos V, Lê KA, Castelli KM, Marshall S, Fayet-Moore F. Balanced carbohydrate ratios are associated with improved diet quality in Australia: a nationally representative cross-sectional study. PLoS One. 2021;16(7):e0253582. 34242252 10.1371/journal.pone.0253582 PMC8270120

[59] Augustin LS , Kendall CW, Jenkins DJ, Willett WC, Astrup A, Barclay AW, Björck I, Brand-Miller JC, Brighenti F, Buyken AE et al. Glycemic index, glycemic load and glycemic response: an international scientific consensus summit from the International Carbohydrate Quality Consortium (ICQC). Nutr Metab Cardiovasc Dis. 2015;25(9):795–815. 26160327 10.1016/j.numecd.2015.05.005

[60] Menday H , Neal B, Wu JHY, Crino M, Baines S, Petersen KS. Use of added sugars instead of total sugars may improve the capacity of the health star rating system to discriminate between core and discretionary foods. J Acad Nutr Diet. 2017;117(12):1921–30..e11. 29173348 10.1016/j.jand.2017.08.013

[61] Comerford KB , Papanikolaou Y, Jones JM, Rodriguez J, Slavin J, Angadi S, Drewnowski A. Toward an evidence-based definition and classification of carbohydrate food quality: an expert panel report. Nutrients. 2021;13(8):2667. 34444826 10.3390/nu13082667 PMC8398407

